# Glucocorticoid‐transactivated TSC22D3 attenuates hypoxia‐ and diabetes‐induced Müller glial galectin‐1 expression via HIF‐1α destabilization

**DOI:** 10.1111/jcmm.15116

**Published:** 2020-03-09

**Authors:** Atsuhiro Kanda, Ikuyo Hirose, Kousuke Noda, Miyuki Murata, Susumu Ishida

**Affiliations:** ^1^ Laboratory of Ocular Cell Biology and Visual Science Department of Ophthalmology Faculty of Medicine and Graduate School of Medicine Hokkaido University Sapporo Japan

**Keywords:** diabetic retinopathy, galectin‐1, glucocorticoid, HIF‐1α, hypoxia, Müller glia, transactivation, TSC22D3

## Abstract

Galectin‐1/*LGALS1*, a newly recognized angiogenic factor, contributes to the pathogenesis of diabetic retinopathy (DR). Recently, we demonstrated that glucocorticoids suppressed an interleukin‐1β‐driven inflammatory pathway for galectin‐1 expression in vitro and in vivo. Here, we show glucocorticoid‐mediated inhibitory mechanism against hypoxia‐inducible factor (HIF)‐1α‐involved galectin‐1 expression in human Müller glial cells and the retina of diabetic mice. Hypoxia‐induced increases in galectin‐1/*LGALS1* expression and promoter activity were attenuated by dexamethasone and triamcinolone acetonide in vitro. Glucocorticoid application to hypoxia‐stimulated cells decreased HIF‐1α protein, but not mRNA, together with its DNA‐binding activity, while transactivating TSC22 domain family member (TSC22D)3 mRNA and protein expression. Co‐immunoprecipitation revealed that glucocorticoid‐transactivated TSC22D3 interacted with HIF‐1α, leading to degradation of hypoxia‐stabilized HIF‐1α via the ubiquitin‐proteasome pathway. Silencing *TSC22D3* reversed glucocorticoid‐mediated ubiquitination of HIF‐1α and subsequent down‐regulation of HIF‐1α and galectin‐1/*LGALS1* levels. Glucocorticoid treatment to mice significantly alleviated diabetes‐induced retinal HIF‐1α and galectin‐1/*Lgals1* levels, while increasing TSC22D3 expression. Fibrovascular tissues from patients with proliferative DR demonstrated co‐localization of galectin‐1 and HIF‐1α in glial cells partially positive for TSC22D3. These results indicate that glucocorticoid‐transactivated TSC22D3 attenuates hypoxia‐ and diabetes‐induced retinal glial galectin‐1/*LGALS1* expression via HIF‐1α destabilization, highlighting therapeutic implications for DR in the era of anti‐vascular endothelial growth factor treatment.

## 1 INTRODUCTION

Diabetic retinopathy (DR), a major retinal microvascular complication in patients with diabetes, is a leading cause of severe visual impairment worldwide. Studies have shown the association of retinal leucocyte infiltration with the pathogenesis of DR, which is now considered as an inflammatory as well as angiogenic disease.^1^ The advanced stage of DR, proliferative DR (PDR), develops fibrovascular proliferation whereby new abnormal blood vessels and fibrous tissues grow on the surface of the retina, resulting in severe complications such as vitreous haemorrhage and traction retinal detachment. Hypoxia promotes pathological neovascularization by up‐regulating the production of angiogenic factors in PDR. Among numerous cytokines and growth factors involved in the pathogenesis of DR, vascular endothelial growth factor (VEGF)‐A is a key mediator for ischaemia‐induced retinal neovascularization and inflammation.^2^, ^3^ Dysfunction of Müller glial cells affected in the ischaemic retina promotes the angiogenic activity of DR as the major source of VEGF‐A overproduction.^4^

Increasing evidence demonstrates the involvement of galectins, a lectin family of β‐galactoside‐binding proteins, with multiple biological events in various cells, tissues and organs.^5^ Galectin‐1, encoded by *lectin, galactoside‐binding, soluble* (*LGALS*)*1* gene, regulates cellular signalling, proliferation and survival in the context of diverse physiological functions,^6^ but has recently proven to play a facilitatory role in hypoxia‐induced pathological angiogenesis in cancer and PDR.^7^, ^8^ Hypoxia‐inducible factor (HIF)‐1α is a known mediator for increased expression of galectin‐1 on top of VEGF‐A.^7^, ^8^ As a regulator for angiogenesis, galectin‐1 has been identified as a VEGF receptor (VEGFR)2 ligand that binds to the *N*‐glycans of VEGFR2 via lectin activity, causing the downstream signal transduction of VEGFR2 in vascular endothelial cells.^7^, ^9^ In addition to the hypoxic induction of galectin‐1, we revealed an interleukin (IL)‐1β‐driven inflammatory pathway to produce galectin‐1 in human Müller glial cells as well as in the retina of diabetic mice.^10^ Activator protein (AP)‐1 is the key transcription factor for the hypoxia‐unrelated *LGALS1* expression in the downstream of IL‐1β receptor‐mediated phosphorylation of phosphatidylinositol‐3 kinase (PI3K)/AKT and extracellular signal‐regulated kinase (ERK)1/2.^10^, ^11^ Recently, we have shown that glucocorticoids inhibit IL‐1β‐induced galectin‐1 expression via dual‐specificity phosphatase (DUSP)1‐dependent and DUSP1‐independent deactivation of AP‐1 signalling (*ie* transactivation and transrepression, respectively) in Müller glial cells.^11^

However, the detailed molecular mechanism of hypoxia‐induced galectin‐1 expression in Müller cells as well as in diabetic retinopathy remains largely unknown especially in terms of glucocorticoid‐mediated regulation. TSC22 domain family member (TSC22D)3, also known as glucocorticoid‐induced leucine zipper, is one of glucocorticoid‐responsive anti‐inflammatory molecules other than DUSP1 and regulates intracellular signalling pathways via HIF‐1α as well as AP‐1.^12^, ^13^ In this study, we demonstrated the regulatory mechanism of TSC22D3/HIF‐1α‐involved galectin‐1 expression in vitro and in vivo, which was further supported by surgical specimens excised from patients with PDR.

## 2 MATERIALS AND METHODS

### 2.1 Cell line and reagents

The human Müller glial cell line Moorfields/Institute of Ophthalmology‐Müller 1 (MIO‐M1) was provided from Dr G. Astrid Limb (UCL Institute of Ophthalmology, London, United Kingdom).^14^ The cells were cultured in DMEM containing 10% fetal bovine serum (Thermo Fisher Scientific). For hypoxic exposure, cells were cultured in a gas mixture composed of 1% O_2_, 5% CO_2_ and 94% N_2_. Aldosterone and streptozotocin were from Sigma‐Aldrich. RU486 and MG132 were from Cayman Chemical. Dexamethasone sodium phosphate, triamcinolone acetonide and actinomycin D were from FUJIFILM Wako Pure Chemical Corporation.

Specific siRNAs against *TSC22D3* (hs.Ri.TSC22D3.13.1), *DUSP1* (hs.Ri.DUSP1.13.3) and a negative control siRNA oligo (DS NC1) were purchased from Integrated DNA Technologies and used at 10 nmol/L.^11^ Cells were transfected with siRNA using Lipofectamine RNAiMAX Reagent (Thermo Fisher Scientific) following the manufacturer's protocols.

### 2.2 Real‐time quantitative PCR (qPCR)

Total RNA isolation was performed from cells using SuperPrep II Cell Lysis & RT Kit for qPCR (TOYOBO) and from tissue samples using TRI reagent (Molecular research centre), as previously described.^7^, ^10^, ^11^, ^15^ The following primers were used: human *LGALS1* (forward 5′‐CGC TAA GAG CTT CGT GCT GAA C‐3′, reverse 5′‐CAC ACC TCT GCA ACA CTT CCA G‐3′), human *HIF1A* (HIF‐1α; forward 5′‐TGC TCA TCA GTT GCC ACT TC‐3′, reverse 5′‐TCC TCA CAC GCA AAT AGC TG‐3′), human *VEGFA* (forward 5′‐CAG ATT ATG CGG ATC AAA CCT CA‐3′; reverse 5′‐CAA GGC CCA CAG GGA TTT TC‐3′), human *TSC22D3* (forward 5′‐ATC TGC AAC CGC AAC ATC GAC C‐3′, reverse 5′‐GCA TAC ATC AGA TGA TTC TTC ACC‐3′), human *DUSP1* (forward 5′‐CTG CCT TGA TCA ACG TCT CA‐3′, reverse 5′‐CTG TGC CTT GTG GTT GTC CT‐3′), human *ACTB* (β‐actin; forward 5′‐CTG GAA CGG TGA AGG TGA CA‐3′, reverse 5′‐ AAG GGA CTT CCT GTA ACA ATG CA‐3′), mouse *Lgals1* (forward 5′‐GTC TCA GGA ATC TCT TCG CTT C‐3′, reverse 5′‐TCC CCG AAC TTT GAG ACA TTC‐3′, probe 5′‐TTC AAT CAT GGC CTG TGG TCT GGT‐3′), mouse *Tsc22d3* (forward 5′‐TCA ATG AGG GCA TCT GCA ACC G‐3′, reverse 5′‐CAT CAG GTG GTT CTT CAC GAG G‐3′), and mouse *Actb* (forward 5′‐CAT CCG TAA AGA CCT CTA TGC CAA C‐3′, reverse 5′‐ATG GAG CCA CCG ATC CAC A‐3′). Real‐time qPCR was performed using the GoTaq qPCR Master mix (Promega), THUNDERBIRD Probe qPCR Mix (TOYOBO), KOD SYBR qPCR Mix (TOYOBO) and StepOne Plus Systems (Thermo Fisher Scientific). Gene expression levels were calculated using the 2^−ddCt^ method, and all experimental samples were normalized with *ACTB* or *Actb* as an internal control.

### 2.3 Enzyme‐linked immunosorbent assay (ELISA)

The protein levels of galectin‐1 in cell lysates and culture supernatants were determined with human galectin‐1 ELISA kit (R&D systems) per the manufacturer's instructions. The optical density was determined using a microplate reader (Sunrise, TECAN).

### 2.4 Immunoblot analyses

Cell extracts were lysed in SDS buffer, a protease inhibitor cocktail (Promega) and a phosphatase inhibitor cocktail (FUJIFILM Wako Pure Chemical Corporation). After quantifying protein concentrations using BCA reagent (Thermo Fisher Scientific), proteins were resolved by SDS‐PAGE (polyacrylamide gel electrophoresis) and transferred to nitrocellulose membrane by electroblotting. Membranes were blocked in TBS containing 5% skim milk and probed with the following primary antibodies: goat anti‐galecint‐1 (1:1000, R&D systems), mouse anti‐TSC22D3 (1:500), mouse anti‐ubiquitin (1:500, Santa Cruz Biotechnology), rabbit anti‐HIF‐1α (1:1000), rabbit anti‐phosphorylated AKT (1:2000), rabbit anti‐AKT (1:2000), rabbit anti‐phosphorylated ERK1/2 (1:2000), rabbit anti‐ERK1/2 (1:2000, Cell signaling technology) and rabbit anti‐β‐actin (1:4000, Medical & Biological Laboratories) antibodies. Horseradish peroxidase‐conjugated anti‐goat, anti‐mouse and anti‐rabbit IgGs (Jackson ImmunoResearch Laboratories) were used as a secondary antibody for chemoluminescence detection. Signals were visualized using a SuperSignal West Pico PLUS Chemiluminescent Substrate (Thermo Fisher Scientific).

### 2.5 Reporter assays

Human *LGALS1* promoter (−500 bp to +67 bp from the *LGALS1* transcription start site)^11^, ^16^, ^17^ was synthesized and sequenced by Integrated DNA Technologies and subcloned into the pGL4 vector (Promega). Luciferase reporter construct containing 3 consensus hypoxia‐responsive elements (HREs) was obtained from Addgene. The pRL‐CMV Renilla luciferase plasmid (Promega) was used as an internal control. The dual‐luciferase reporter assays system (Promega) was used to measure the activity of firefly and Renilla luciferase. Cells were transfected with plasmid DNA was transfected using Lipofectamine LTX with Plus Reagent (Thermo Fisher Scientific) following the manufacturer's protocols.

### 2.6 Chromatin immunoprecipitation‐qPCR (ChIP‐qPCR)

Assays were performed using the SimpleChIP Enzymatic Immunoprecipitation Chromatin IP Kit (Cell Signaling Technology) according to the manufacturer's protocols. Chromatin was immunoprecipitated with mouse anti‐HIF‐1α (Novus Biologicals) antibody. Normal mouse IgG (R&D systems) was used as control. Thereafter, chromatin immunoprecipitates were evaluated by real‐time qPCR using the primers specific for the previously described HRE sites in the *LGALS1* promoter region (forward 5′‐ CCC AGC CTT TCT TTA GCC TTC C ‐3′, reverse 5′‐ GAT GAT GAG CTA GGC CCA CAA G ‐3′)^8^ together with 2% input DNA as reference samples. Real‐time qPCR was performed using KOD SYBR qPCR Mix (TOYOBO) and StepOne Plus Systems (Thermo Fisher Scientific). ChIP‐qPCR signals were calculated as percentage of input.

### 2.7 Co‐immunoprecipitation (co‐IP)

Cells were homogenized in lysis buffer (1% NP‐40 in PBS) with a protease inhibitor cocktail (Promega) and a phosphatase inhibitor cocktail (FUJIFILM Wako Pure Chemical Corporation). After sonicated cell extracts were preabsorbed with Dynabeads Protein G (Thermo Fisher Scientific), the cell extracts were incubated with antibodies and Dynabeads Protein G overnight at 4°C with gentle mixing. The beads were washed with the lysis buffer and suspended in SDS sample buffer. The eluted proteins were analysed by immunoblot analysis.

### 2.8 Animals and induction of diabetes

C57BL/6J mice were obtained from CLEA Japan (Tokyo, Japan). All animal experiments were conducted in accordance with the ARVO (Association of Research in Vision and Ophthalmology) Statement for the Use of Animals in Ophthalmic and Vision Research and approved by the Ethics Review Committee for Animal Experimentation of Hokkaido University. Procedures for murine model of streptozotocin‐induced diabetes were described in our previous reports.^7^, ^10^, ^11^ At 2 months after induction of diabetes, dexamethasone and triamcinolone acetonide (50 pmol/eye for each) were injected into the vitreous cavity.

### 2.9 Human surgical samples

During vitrectomy for traction retinal detachment, 5 fibrovascular tissues were excised from PDR eyes and used for immunohistochemistry. This study was conducted in accordance with the tenets of the Declaration of Helsinki and after receiving approval from the institutional review board of Hokkaido University Hospital. Written informed consent was obtained from all patients after explanation of the purpose and procedures of this study.

### 2.10 Immunofluorescence microscopy

Immunofluorescence analyses were performed as described previously.^7^, ^10^, ^11^, ^15^ Serial sections were incubated with the following primary antibodies: goat anti‐galectin‐1 (1:100, R&D systems), rabbit anti‐glial fibrillary acidic protein (1:200, GFAP; Leica), mouse anti‐HIF‐1α (1:100, Novus Biologicals), rabbit anti‐HIF‐1α (1:100, Cell signaling technology) and mouse anti‐TSC22D3 (1:50, Santa Cruz Biotechnology) antibodies. Secondary antibodies for fluorescent detection were AlexaFluor 488 and 546 (Thermo Fisher Scientific). Nuclei were counterstained with DAPI (4′,6‐diamidino‐2‐phenylindole), and sections were visualized under a Keyence BZ‐9000 (Keyence).

### 2.11 Statistical analyses

All the results are expressed as the mean ± SEM (standard error of the mean). Student's *t* test was used for statistical comparison between groups, and one‐way analysis of variance (ANOVA) followed by the Tukey‐Kramer method as a post hoc test was used for multiple comparison procedures. Differences between means were considered statistically significant when *P* values were <.05.

## 3 RESULTS

### 3.1 Glucocorticoid‐mediated suppression of hypoxia‐induced galectin‐1/*LGALS1* expression in human Müller glial cells

**Figure 1:**
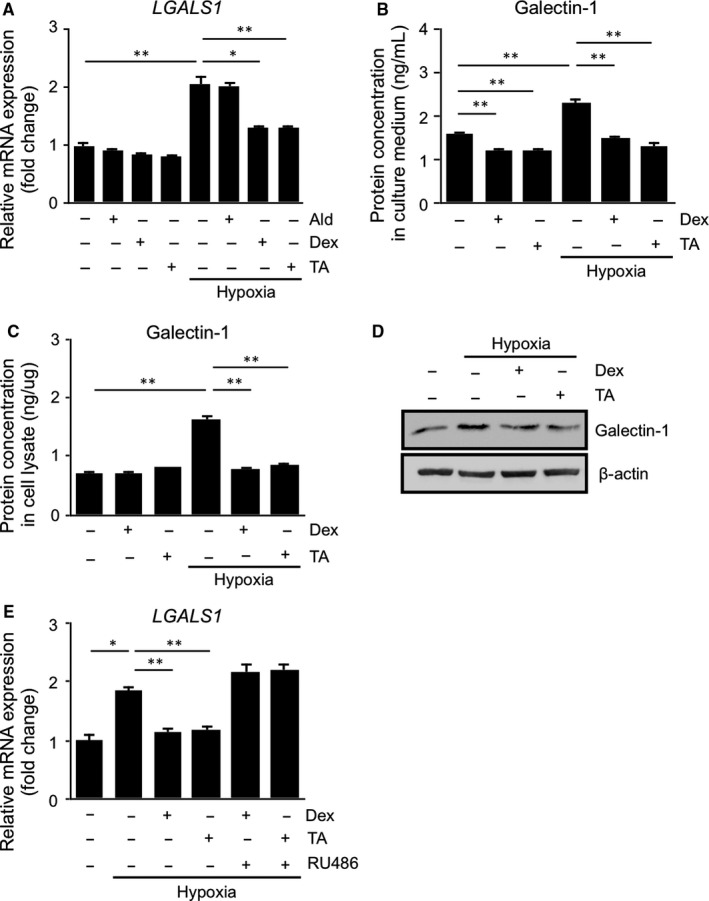
Glucocorticoid‐mediated suppression of hypoxia‐induced galectin‐1/*LGALS1* expression in human Müller glial cells. (A) Müller glial cells were pretreated with aldosterone (Ald, 1 μmol/L), dexamethasone (Dex, 1 μmol/L) or triamcinolone acetonide (TA, 1 μmol/L) for 30 min before culture in hypoxia (1% O_2_) for 24 h, and *LGALS1* gene expression levels were analysed. (B‐D) Müller glial cells were pretreated with Dex (1 μmol/L) or TA (1 μmol/L) for 30 min before culture in hypoxia (1% O_2_) for 24 h, and galectin‐1 protein expression levels in culture medium (B) and cell lysate (C, D) were analysed by ELISA (B, C) and immunoblot analysis (D). (E) Müller glial cells were pretreated with the glucocorticoid receptor antagonist RU486 (1 μmol/L) for 30 min before culture with Dex (1 μmol/L) and TA (1 μmol/L) in hypoxia (1% O_2_) for 24 h, and *LGALS1* gene expression levels were analysed. *P < .05, **P < .01, n = 6 per group

HIF‐1α protein, an oxygen‐dependent transcriptional activator, controls various gene expression.^18^ We and others demonstrated that hypoxia‐up‐regulated galectin‐1/*LGALS1* expression in retinal tissues and cell lines through HIF‐1α.^7^, ^8^, ^10^ Given that glucocorticoids suppress hypoxia‐induced gene expression via reducing the protein levels and DNA‐binding activity of HIF‐1α,^19^ we investigated whether administration of glucocorticoids alters hypoxia‐induced galectin‐1 production in human Müller glial cells. Hypoxic stimulation significantly up‐regulated *LGALS1* mRNA expression, which was abolished by both of the glucocorticoids dexamethasone (*P* < .05) and triamcinolone acetonide (*P* < .01), but not by the mineralocorticoid aldosterone (Figure 1A). We further analysed the protein levels of galectin‐1 in the culture medium and cell lysate of Müller glial cells under hypoxia. In accordance with the gene expression data, the protein levels of galectin‐1 in the culture medium (Figure 1B) and cell lysate (Figure 1C,D), elevated by hypoxia, were reduced by dexamethasone and triamcinolone acetonide (*P* < .01 for all). Moreover, the suppressive effects of dexamethasone and triamcinolone acetonide on *LGALS1* mRNA expression were reversed by pretreatment with the glucocorticoid receptor antagonist RU486 (Figure 1E), indicating a significant contribution of glucocorticoids and the glucocorticoid receptor to hypoxia‐induced galectin‐1/*LGALS1* expression in Müller glial cells.

### 3.2 Glucocorticoid‐mediated reduction of HIF‐1α protein and DNA‐binding activity with no impact on *LGALS1* mRNA stability

**Figure 2:**
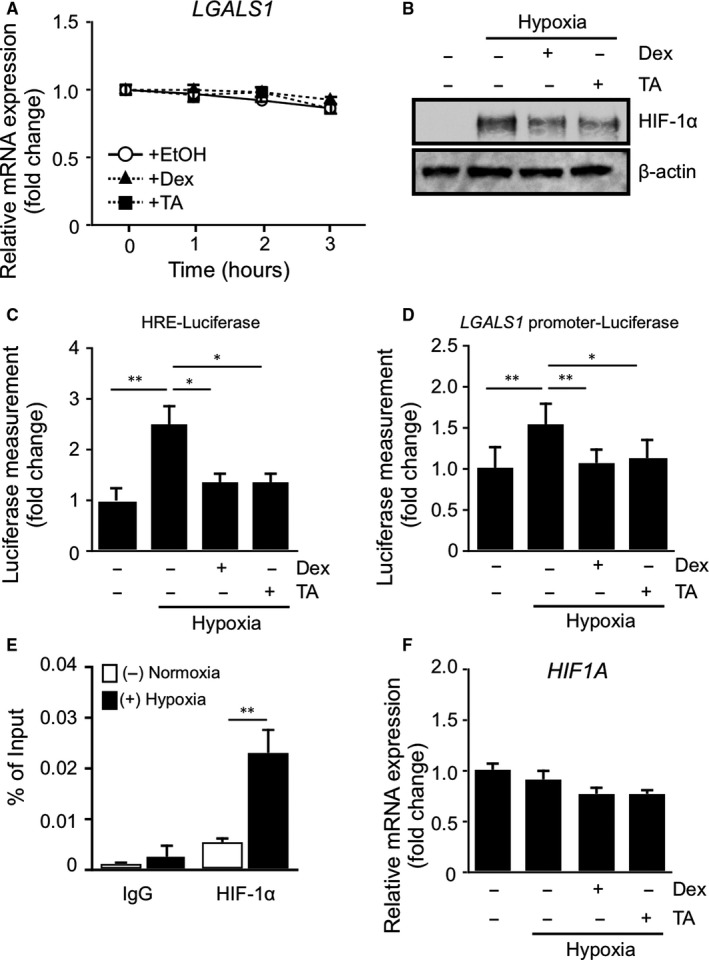
Glucocorticoid‐mediated reduction of HIF‐1α protein and DNA‐binding activity with no impact on *LGALS1* mRNA stability. (A) After Müller glial cells were cultured in hypoxia (1% O_2_) for 24 h, the transcription inhibitor actinomycin D (2.5 μg/mL) with or without dexamethasone (Dex, 1 μmol/L) or triamcinolone acetonide (TA, 1 μmol/L) was added and cells were harvested at the indicated times. RNA was extracted for real‐time qPCR analysis of *LGALS1*. (B) Müller glial cells were pretreated with Dex (1 μmol/L) or TA (1 μmol/L) for 30 min before culture in hypoxia (1% O_2_) for 24 h, and HIF‐1α protein expression levels were analysed. (C, D) Müller glial cells were transfected with the control reporter pRL‐CMV, together with consensus HRE‐luciferase reporter (C) or human *LGALS1* promoter‐luciferase reporter (D). Transfected Müller glial cells were pretreated with Dex (1 μmol/L) or TA (1 μmol/L) for 30 min before culture in hypoxia (1% O_2_) for 24 h and assayed for luciferase activities. (E) Müller glial cells were cultured in hypoxia (1% O_2_) for 1 h before harvest of samples. Binding of HIF‐1α to HREs in the *LGALS1* promoter region was analysed by ChIP‐qPCR. (F) Müller glial cells were pretreated with Dex (1 μmol/L) or TA (1 μmol/L) for 30 min before culture in hypoxia (1% O_2_) for 24 h, and *HIF1A* gene expression levels were analysed. *P < .05, **P < .01, n = 4‐6 per group

In response to its ligands, the glucocorticoid receptor acts as a DNA‐binding transcription factor^20^ in addition to a negative regulator of mRNA stability,^21^ thus controlling diverse cellular functions. Previous studies demonstrated that application of glucocorticoids to various cell lines rapidly induced the mRNA decay of target genes related to inflammation.^22^, ^23^, ^24^ To investigate the mechanism by which glucocorticoids down‐regulate *LGALS1* expression, we next addressed its effect on *LGALS1* mRNA stability. Cells were cultured under hypoxia for 24 hours and then incubated with actinomycin D, a transcription inhibitor, in the presence or absence of glucocorticoids. Treatment with glucocorticoids did not change *LGALS1* mRNA levels (Figure 2A), suggesting no impact on *LGALS1* mRNA stability. Moreover, to investigate the involvement of HIF‐1α in glucocorticoid‐mediated galectin‐1 suppression, we studied the effect of glucocorticoids on HIF‐1α protein and its DNA‐binding activity. HIF‐1α protein levels (Figure 2B) and transcriptional activities in plasmids HRE‐luciferase (Figure 2C) and *LGALS1* promoter‐luciferase (Figure 2D) were augmented by hypoxic stimulation, all of which were reversed by glucocorticoids. Consistently, ChIP‐qPCR revealed that binding of HIF‐1α to HREs in the *LGALS1* promoter region was significantly up‐regulated under hypoxia (Figure 2E), indicating a significant role of HREs in the *LGALS1* promoter region in hypoxia‐induced galectin‐1/*LGALS1* expression in Müller glial cells. Next, we confirmed the absence of changes in *HIF1A* mRNA expression under hypoxia up to 48 hours, despite time‐dependent responsiveness of HIF‐1α‐regulated *LGALS1* and *VEGFA* transcripts (Figure S1A‐C). We additionally found that glucocorticoid treatment resulted in no difference in *HIF1A* mRNA expression in human Müller glial cells (Figure 2F). These results suggested that glucocorticoid‐mediated reduction of HIF‐1α protein levels led to down‐regulation of hypoxia‐induced galectin‐1/*LGALS1* expression, while *LGALS1* mRNA was not destabilized by glucocorticoids.

### 3.3 Glucocorticoid‐transactivated TSC22D3 interaction with and ubiquitination of HIF‐1α leading to suppression of hypoxia‐induced galectin‐1/*LGALS1* expression

**Figure 3:**
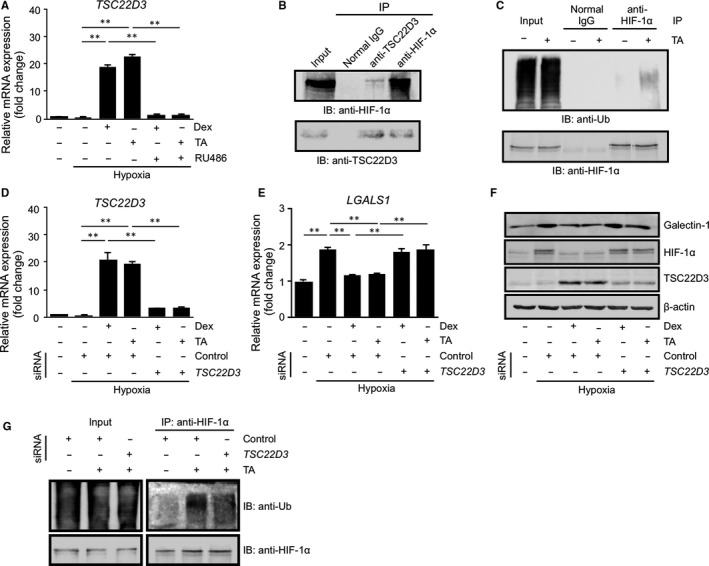
Glucocorticoid‐transactivated TSC22D3 interaction with and ubiquitination of HIF‐1α leading to suppression of hypoxia‐induced galectin‐1/*LGALS1* expression. (A) Müller glial cells were pretreated with the glucocorticoid receptor antagonist (RU486, 1 μmol/L) for 30 min before culture with dexamethasone (Dex, 1 μmol/L) or triamcinolone acetonide (TA, 1 μmol/L) in hypoxia (1% O_2_) for 24 h, and *TSC22D3* gene expression levels were analysed. (B) After hypoxic stimulation (1% O_2_) for 24 h, co‐IP of human Müller glial cell extracts using anti‐TSC22D3 and anti‐HIF‐1α antibodies was performed, followed by immunoblot analyses for HIF‐1α and TSC22D3. (C) Müller glial cells were incubated in hypoxia (1% O_2_) with or without TA in the presence of the proteasome inhibitor MG132 (10 μmol/L) for 24 h. After co‐IP of cell extracts with anti‐HIF‐1α antibody, ubiquitinated HIF‐1α was detected using anti‐ubiquitin (Ub) antibody in TA‐treated cell extracts. (D, E) *TSC22D3* (D) and *LGALS1* (E) mRNA expression levels in human Müller glial cells exposed to control‐ or *TSC22D3*‐siRNA combined with Dex (1 μmol/L) or TA (1 μmol/L) for 30 min before culture in hypoxia (1% O_2_) for 24 h. (F) Galectin‐1, HIF‐1α and TSC22D3 protein expression levels in human Müller glial cells exposed to control‐ or *TSC22D3*‐siRNA combined with Dex (1 μmol/L) or TA (1 μmol/L) for 30 min before culture in hypoxia (1% O_2_) for 24 h. (G) Müller glial cells transfected with control‐ or *TSC22D3*‐siRNA were incubated in hypoxia (1% O_2_) with or without TA in the presence of MG132 (10 μmol/L) for 24 h. After co‐IP of cell extracts with anti‐HIF‐1α antibody, ubiquitinated HIF‐1α was detected using anti‐Ub antibody. *P < .05, **P < .01, n = 6 per group

Numerous glucocorticoid‐transactivated genes play critical roles in the anti‐inflammatory action of glucocorticoids.^21^ To understand the molecular mechanism involved in glucocorticoid‐mediated deactivation of the HIF‐1 pathway in Müller glial cells, we examined the expression of TSC22D3, a glucocorticoid‐responsive molecule that interacts with intracellular signalling proteins so as to regulate the transcription of inflammation‐related genes.^12^ Administration of glucocorticoids to Müller glial cells significantly up‐regulated *TSC22D3* mRNA expression (*P* < .01), which was abolished by pretreatment with RU486 (*P* < .01) (Figure 3A). TSC22D3 was shown to directly binds to and degrades HIF‐1α protein through the ubiquitin‐proteasome pathway in human distal lung epithelial cells.^13^ To explore the involvement of TSC22D3 in the glucocorticoid‐mediated down‐regulation of HIF‐1α protein, co‐IP experiments followed by immunoblot analyses were performed with triamcinolone acetonide‐treated Müller glial cells using antibodies against TSC22D3 and HIF‐1α. IP with anti‐TSC22D3 antibody from triamcinolone acetonide‐treated cell extracts exhibited that TSC22D3 could pull down HIF‐1α, while reverse IP for HIF‐1α detected TSC22D3 (Figure 3B). Next, we carried out a ubiquitination assay for HIF‐1α in Müller glial cells treated with triamcinolone acetonide. To block HIF‐1α protein degradation, cells were incubated with the proteasome inhibitor MG132. As shown in Figure 3C, ubiquitination of HIF‐1α protein was enhanced by triamcinolone acetonide application, demonstrating that glucocorticoid‐mediated HIF‐1α protein down‐regulation (Figure 2B) was achieved via the ubiquitin‐proteasome pathway. To validate the involvement of TSC22D3 in the suppression of hypoxia‐induced galectin‐1/*LGALS1* expression, we preformed siRNA experiments against *TSC22D3* in Müller glial cells. Gene expression results proved the siRNA‐mediated potent inhibition of *TSC22D3* mRNA levels (*P* < .01, Figure 3D). Interestingly, silencing *TSC22D3* almost completely cancelled glucocorticoid‐mediated decline of *LGALS1* transcripts (*P* < .05, Figure 3E). Moreover, immunoblot data confirmed the reverse effect of *TSC22D3* knockdown on glucocorticoid‐mediated reduction of galectin‐1 and HIF‐1α protein levels in Müller glial cells (Figure 3F). Importantly, silencing *TSC22D3* reduced the glucocorticoid‐mediated ubiquitination of HIF‐1α protein (Figure 3G). These results indicated that transactivated TSC22D3 interacted with and degraded HIF‐1α protein through the ubiquitin‐proteasome pathway, thus inhibiting HIF‐1α‐driven galectin‐1/*LGALS1* expression in Müller glial cells.

Additionally, we examined whether DUSP1 (also known as mitogen‐activated protein kinase phosphatase‐1) contributes to the molecular mechanism of glucocorticoid‐mediated galectin‐1/*LGALS1* down‐regulation. We recently showed that glucocorticoid‐transactivated DUSP1 mitigated IL‐1β‐induced galectin‐1/*LGALS1* expression by reversing the phosphorylation of AKT and ERK1/2.^11^ Administration of glucocorticoids to Müller glial cells significantly enhanced the expression of *DUSP1* (Figure S2A), whereas silencing *DUSP1* did not prevent the glucocorticoid‐mediated suppression of hypoxia‐induced galectin‐1/*LGALS1* expression (Figure S2B). To further support the results, immunoblotting also showed that hypoxia did not change the protein levels of phosphorylated and total forms of AKT and ERK1/2 (Figure S2C). These data indicated that transactivated DUSP1 was not involved in glucocorticoid‐mediated down‐regulation of hypoxia‐induced galectin‐1/*LGALS1* expression in Müller glial cells.

### 3.4 Glucocorticoid‐mediated inhibition of diabetes‐induced retinal galectin‐1 and HIF‐1α together with transactivation of TSC22D3 in mice

**Figure 4:**
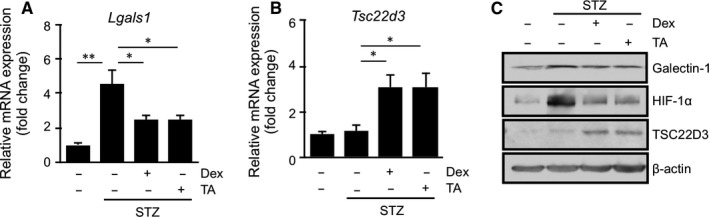
Glucocorticoid‐mediated inhibition of diabetes‐induced retinal galectin‐1 and HIF‐1α together with transactivation of TSC22D3 in mice. (A‐C) Retinal *Lgals1* (A) and *Tsc22d3* (B) expression in mice with streptozotocin (STZ)‐induced diabetes at 2 mo. Dexamethasone (Dex, 50 pmol/eye) or triamcinolone acetonide (TA, 50 pmol/eye) were injected intravitreally to STZ mice, followed by mRNA (A, B) and protein (C) expression analyses after 24 h. (C) Immunoblot analyses for galectin‐1, TSC22D3 and HIF‐1α in the retina of diabetic mice treated with Dex or TA. *P < .05, **P < .01, n = 6‐8 per group

Given that the HIF‐1 pathway is activated in the retina of mice with streptozotocin‐induced diabetes,^25^ we performed in vivo experiments to further verify the inhibitory effect of glucocorticoids on hypoxia‐induced galectin‐1/*LGALS1* expression in vitro. We previously reported diabetes‐induced up‐regulation of galectin‐1 protein expression mainly in Müller glial cells in the murine retina.^10^ Consistent with our previous reports,^7^, ^10^, ^11^ galectin‐1/*Lgals1* expression in the retina of mice with streptozotocin‐induced diabetes at 2 months was significantly higher than that in controls and was abolished by intravitreal injection of glucocorticoids (Figure 4A,C). Moreover, both mRNA and protein levels of *Tsc22d3* in the diabetic retina was induced by glucocorticoid treatment (Figure 4B,C). We also verified that treatment with glucocorticoids reduced diabetes‐induced HIF‐1α protein up‐regulation (Figure 4C). These results suggested that induction of diabetes activated the HIF‐1 pathway followed by retinal Müller glial galectin‐1 up‐regulation, both of which were suppressed by glucocorticoid‐transactivated TSC22D3.

### 3.5 Tissue co‐localization of HIF‐1α and galectin‐1 in glial cells in the epiretinal fibrovascular tissue excised from eyes of patients with PDR

**Figure 5:**
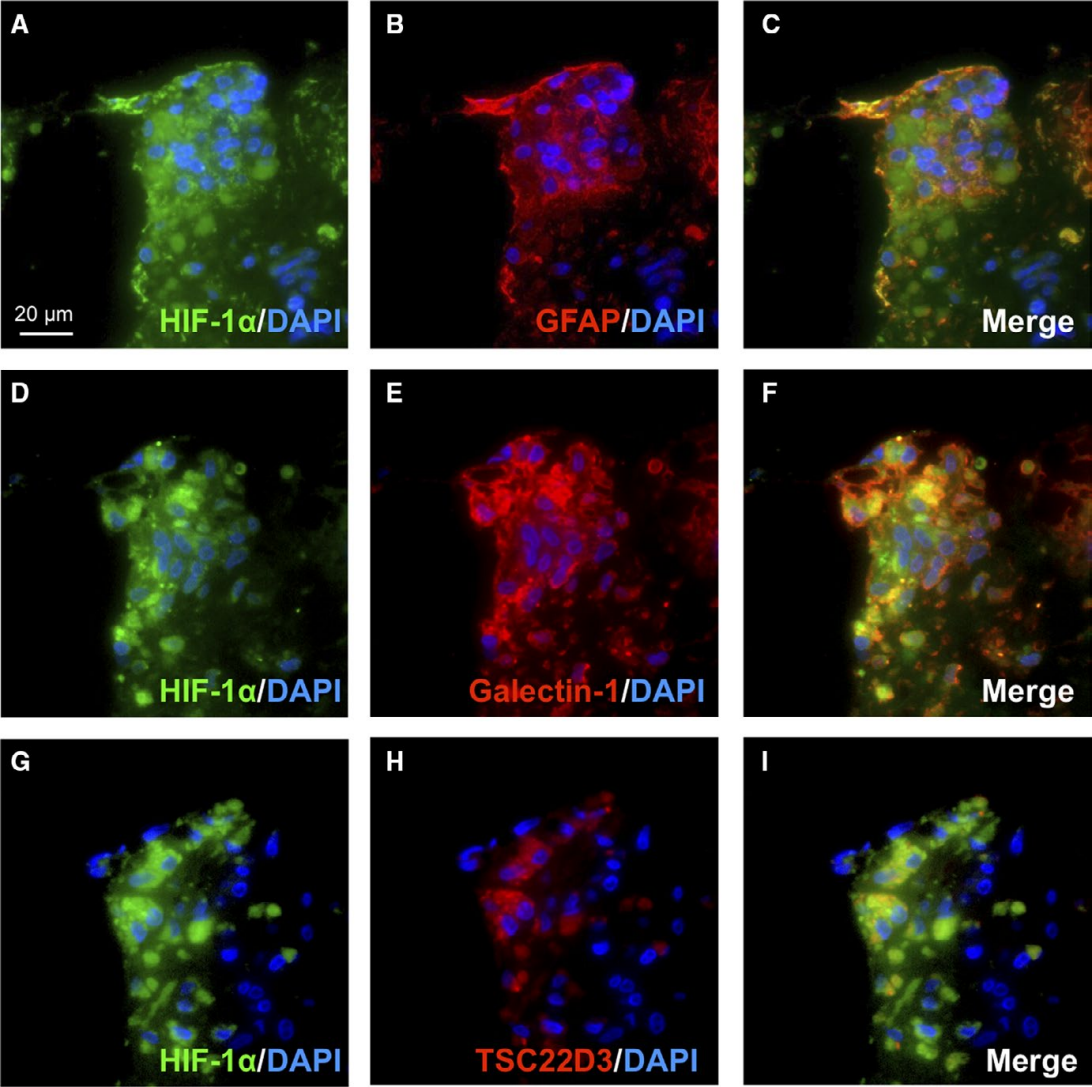
Tissue co‐localization of HIF‐1α and galectin‐1 in glial cells in the epiretinal fibrovascular tissue excised from eyes of patients with PDR. (A‐C) Double labelling of HIF‐1α (*green*) and GFAP (*red*) with DAPI (*blue*) counterstaining in PDR fibrovascular tissues. (D‐F) Double labelling of HIF‐1α (*green*) and galctin‐1 (*red*) with DAPI (*blue*) counterstaining. (G‐I) Double labelling of HIF‐1α (*green*) and TSD22D3 (*red*) with DAPI (*blue*) counterstaining. Scale bar = 20 μm

GFAP‐positive glial cells were shown to exhibit higher HIF‐1α immunoreactivity in surgically removed epiretinal tissues from patients with PDR than non‐diabetic controls with idiopathic epiretinal membrane.^26^ Previously, we demonstrated co‐localization of galectin‐1 and the glucocorticoid receptor in glial cells in the fibrovascular tissue taken from eyes of PDR patients.^11^ Similarly, we performed immunofluorescence analyses on surgically removed PDR fibrovascular tissues to investigate the tissue localization of HIF‐1α and galectin‐1 in glial cells. In serial sections of PDR patient specimens, GFAP‐positive glial cells expressed HIF‐1α (Figure 5A‐C), which co‐localized with galectin‐1 (Figure 5D‐F). In contrast, HIF‐1α‐positive glial cells exhibited only partial immunoreactivity for TSC22D3 (Figure 5G‐I). These findings suggested a significant contribution of HIF‐1α to the expression of galectin‐1 in Müller glial cells migrating into the proliferative tissue in human PDR.

## 4 DISCUSSION

**Figure 6:**
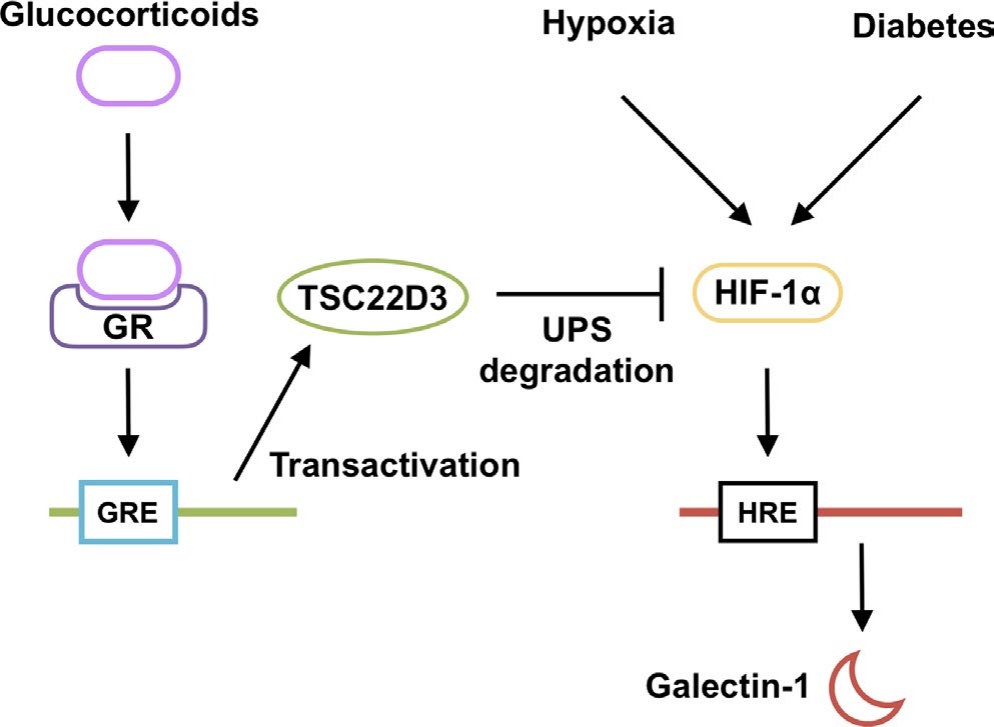
A schema showing that glucocorticoid‐transactivated TSC22D3 suppresses hypoxia‐ and diabetes‐induced galectin‐1 expression through HIF‐1α destabilization. Glucocorticoid‐bound glucocorticoid receptor (GR) transactivates TSC22D3 via glucocorticoid response element (GRE), causing ubiquitin‐proteasome system (UPS)‐mediated degradation of HIF‐1α, which is otherwise stabilized by hypoxia and diabetes for the induction of Müller glial galectin‐1 expression

Glucocorticoids have been implicated in a broad range of biochemical and physiological functions including inflammation, immunity, metabolism, and development.^27^ Recently, we proposed the suppressive effect of glucocorticoids on IL‐1β‐induced Müller glial galectin‐1/*LGALS1* expression through DUSP1 transactivation followed by deactivation of AP‐1 signalling.^11^ Our present study exhibited novel data regarding the glucocorticoid‐mediated down‐regulation of hypoxia‐ and diabetes‐induced galectin‐1 expression, which resulted from an entirely different pathway than the IL‐1β/AP‐1 axis impeded by glucocorticoid‐transactivated DUSP1. Hypoxia‐induced galectin‐1/*LGALS1* expression in Müller glial cells was significantly attenuated by treatment with glucocorticoids dexamethasone and triamcinolone acetonide, and this inhibitory effect was eliminated by pretreatment with the glucocorticoid receptor antagonist (Figure 1). Glucocorticoids reduced the hypoxia‐up‐regulated levels of HIF‐1α protein and its DNA‐binding activity, together with no impact on *LGALS1* mRNA stability (Figure 2). Treatment with glucocorticoids increased the expression of TSC22D3, which then bound to HIF‐1α protein followed by its degradation via the ubiquitin‐proteasome pathway. Silencing *TSC22D3* prevented glucocorticoid‐mediated ubiquitination of HIF‐1α and subsequent down‐regulation of HIF‐1α protein and galectin‐1/*LGALS1* levels in Müller glial cells (Figure 3). In vivo, glucocorticoids injected into murine eyes decreased diabetes‐induced retinal galectin‐1 and TSC22D3 expression and HIF‐1α protein up‐regulation (Figure 4). Importantly, fibrovascular tissues collected from PDR patients demonstrated the co‐localization of HIF‐1α and galectin‐1 in glial cells partially positive for TSC22D3 (Figure 5). These results suggested that glucocorticoid‐transactivated TSC22D3 suppressed hypoxia‐ and diabetes‐induced galectin‐1 expression via destabilization of HIF‐1α protein (Figure 6).

Two HREs are located at position 441 bp to 423 bp upstream of the transcriptional start site of *LGALS1* gene, and are crucial for HIF‐1α‐regulated expression.^8^ We previously reported a significant up‐regulation of *LGALS1* in various retinal cells cultured under hypoxia.^7^ HIF‐1α, a transcription factor, plays critical roles in mammalian development and in the pathogenesis of many diseases, and activates the transcription of various genes controlling adaptive responses to hypoxia.^18^ In normoxia, proline residues within the oxygen‐dependent degradation domain of HIF‐1α are hydroxylated by proline hydroxylase, which triggers interaction with the von Hippel‐Lindau tumour suppressor protein (pVHL) and ubiquitin‐mediated protein degradation of HIF‐1α. Under hypoxic conditions, however, proline hydroxylase loses its enzymatic activity, leading to the stable and functional state of HIF‐1α protein escaping from degradation and then achieving translocation into the nucleus to bind with the HREs in the promoter of hypoxia‐responsive genes. In addition to hypoxic conditions, HIF‐1α activity is also modulated by various signal transduction pathways including PI3K/AKT and mitogen‐activated protein kinase (MAPK)/ERK cascades.^28^, ^29^ Activation of these signalling molecules stimulates the HIF‐1 pathway by promoting the phosphorylation of HIF‐1α in response to hypoxia. We observed that HIF‐1α protein levels significantly up‐regulated in Müller glial cells under hypoxia; however, the expression of *HIF1A* mRNA and phosphorylated AKT and ERK1/2 levels did not change (Figure 2F; Figures S1A and S2C). The hypoxia‐induced activation of HIF‐1α in Müller glial cells is thought to result from enhancement of protein stability, but not transcription or phosphorylation via other signalling pathways.

Accumulating evidence has revealed that several proteins including proline hydroxylase, pVHL, and TSC22D3 interacted with HIF‐1α and reduce its protein stabilization.^30^ TSC22D3, identified as a glucocorticoid‐transactivated gene that functions as a transcriptional regulator, is known to promote the anti‐inflammatory, immunosuppressive, and anti‐proliferative actions of glucocorticoids in various cells.^21^, ^31^ Overexpression of retinal TSC22D3 protected retinal neurons from light‐induced degeneration and lipopolysaccharide‐induced inflammation.^32^, ^33^ TSC22D3 mainly exerts its effects by homo‐ or hetero‐dimerization with specific partner proteins, including transcription factors such as AP‐1, Raf‐1 and Ras, and regulates the expression of target genes at the transcription level.^12^ Recently, TSC22D3 was shown to bind with HIF‐1α and cause its degradation through the ubiquitin‐proteasome pathway.^13^ Consistently, we showed that TSC22D3 interacted with HIF‐1α and increased ubiquitination of HIF‐1α protein in Müller glial cells, serving as a negative regulator of HIF‐1α (Figure 3B,G). Moreover, hypoxia did not alter the activation of IL‐1β‐induced PI3K/AKT and MAPK/ERK1/2 signalling pathways (Figure S2C), both of which were shown to be suppressed by glucocorticoid‐transactivated DUSP1.^11^ All these data supported our conclusion indicating an important role of TSC22D3 in HIF‐1α destabilization in Müller glial cells treated with glucocorticoids.

Several inflammation‐related molecules, such as cyclooxygenase‐2, interferon‐β and tumour necrosis factor‐α, were shown to be swiftly down‐regulated by glucocorticoids at the post‐transcriptional level, because these mRNAs have adenylate/uridylate‐rich elements (AREs) in their untranslated regions.^22^, ^23^, ^24^, ^34^ Glucocorticoids rapidly induce the protein production of tristetraprolin, an ARE‐binding protein also known as zinc finger protein 36 homolog, which recruits 5′‐3′ exoribonuclease 1 causing rapid degradation of target mRNAs.^35^, ^36^, ^37^ We checked the presence of AREs in the untranslated regions of *LGALS1* mRNA using multiple database sites (AREsite2, http://rna.tbi.univie.ac.at/AREsite2/welcome; RegRNA 2.0, http://regrna2.mbc.nctu.edu.tw)^38^, ^39^; however, we could not find any predicted AREs (data not shown). Indeed, treatment with glucocorticoids did not affect *LGALS1* mRNA stability (Figure 2A). These negative results on mRNA degradation, in concert with negligible impact of DUSP1 (Figure S2), supported the data showing that glucocorticoid‐transactivated TSC22D3 was the major pathway to suppress hypoxia‐induced galectin‐1/*LGALS1* expression (Figure 3E,F) via reducing HIF‐1α protein stability in Müller glial cells (Figure 3G).

Reasonably, the currently identified mechanism of suppression would also apply to not only galectin‐1 but also several other HIF‐1α downstream targets responsible for the pathogenesis of DR. Such molecules governed by the HIF‐1 pathway in Müller glial cells include VEGF‐A, intercellular adhesion molecule‐1, and matrix metalloproteinase‐2,^40^, ^41^ all of which promote the inflammatory and angiogenic activity of DR.^42^, ^43^, ^44^ Indeed, glucocorticoids significantly suppressed hypoxia‐induced *VEGFA* expression via the glucocorticoid receptor (Figure S3A), and the mechanism of action depended on TSC22D3 (Figure S3B), which totally mirrored the current results on hypoxia‐induced *LGALS1* expression (Figures 1E and 3E). In the era of anti‐VEGF therapy with ranibizumab and aflibercept, both of which are the gold standard for the management of DR, treatment with glucocorticoids has also been still efficacious,^45^, ^46^, ^47^, ^48^ given that retinal physicians actually encounter anti‐VEGF refractory cases in clinical practice. Aflibercept, a recombinant glycoprotein, is composed of the ligand‐binding domains of VEGFR1 and VEGFR2 fused to the Fc region of human immunoglobulin G.^49^ Previously, we reported the neutralizing efficacy of aflibercept against galectin‐1 as well as VEGF‐A^7^; however, aflibercept does not block intracellular pathways for the production of galectin‐1 or VEGF‐A. Since anti‐inflammatory and ‐angiogenic mechanisms exerted by glucocorticoids and anti‐VEGF drugs are thus theoretically different, their combination therapy can potentially provide additional benefits for DR. Indeed, Protocol U by the Diabetic Retinopathy Clinical Research Network revealed that the combination therapy with ranibizumab plus dexamethasone significantly reduced retinal thickness (*ie* ameliorate diabetic macular oedema) compared with the ranibizumab monotherapy, despite no difference in vision between the arms.^50^ Many questions remain, however, regarding glucocorticoid use in conjunction with anti‐VEGF agents, including the appropriate combination of agents, the timing of therapeutic transition, and the outcomes of such protocols.

Our previous^11^ and current findings revealed that detailed molecular mechanisms by glucocorticoids suppressed diabetes (*ie* inflammation and hypoxia)‐induced galectin‐1 expression via transactivation of DUSP‐1 and TSC22D3, respectively. Since the involvement of inflammation and hypoxia varies in degree depending on the pathogenic stages of DR, glucocorticoids, which can utilize both DUSP‐1 and TSC22D3, are theorized to ensure the advantage of versatility for their clinical use. Even in the golden age of anti‐VEGF treatment, this study focusing on the TSC22D3‐dependent regulation of HIF‐1α may still provide the therapeutic implications of glucocorticoid drugs for the long‐term management of DR, a chronic and multifactorial disease in nature, because the HIF‐1 pathway stimulates the gene expression of multiple inflammatory and angiogenic factors including VEGF‐A on top of galectin‐1.

## CONFLICT OF INTEREST

The authors declare no competing financial or non‐financial interests.

## AUTHOR CONTRIBUTION

AK designed research; AK, IH, KN and MM performed the experiments; AK and IH analysed the data; AK and SI wrote the paper; and all authors approved the final version submitted for publication.

## Supporting information

Fig S1‐S3

## Data Availability

The data that support the findings of this study are available from the corresponding author upon reasonable request.
