# Supplementary material for: Glucocorticoid‐transactivated TSC22D3 attenuates hypoxia‐ and diabetes‐induced Müller glial galectin‐1 expression via HIF‐1α destabilization
Source: J Cell Mol Med. 2020 Mar 9;24(8):4589–99. doi: 10.1111/jcmm.15116 (PMC7176855; doi:10.1111/jcmm.15116)
Supplement: Supplementary file 1 — Fig S1‐S3 [file JCMM-24-4589-s001.pdf]

Supplementary Figure 1

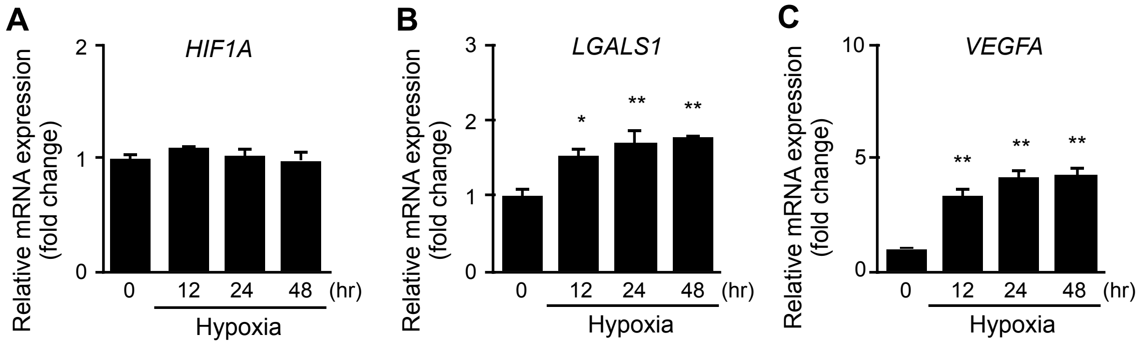

Supplementary Figure S1. Absence of changes in Müller glial expression levels of *HIF1A,* but not *LGALS1* or *VEGFA*, during hypoxia

(A-C) Müller glial cells were cultured in hypoxia (1% O_2) up to 48 hours, and *HIF1A* (A), *LGALS1* (B) and *VEGFA* (C) gene expression levels were analyzed. $*p < 0.05$, $**p < 0.01$, n = 6 per group.

Supplementary Figure 2

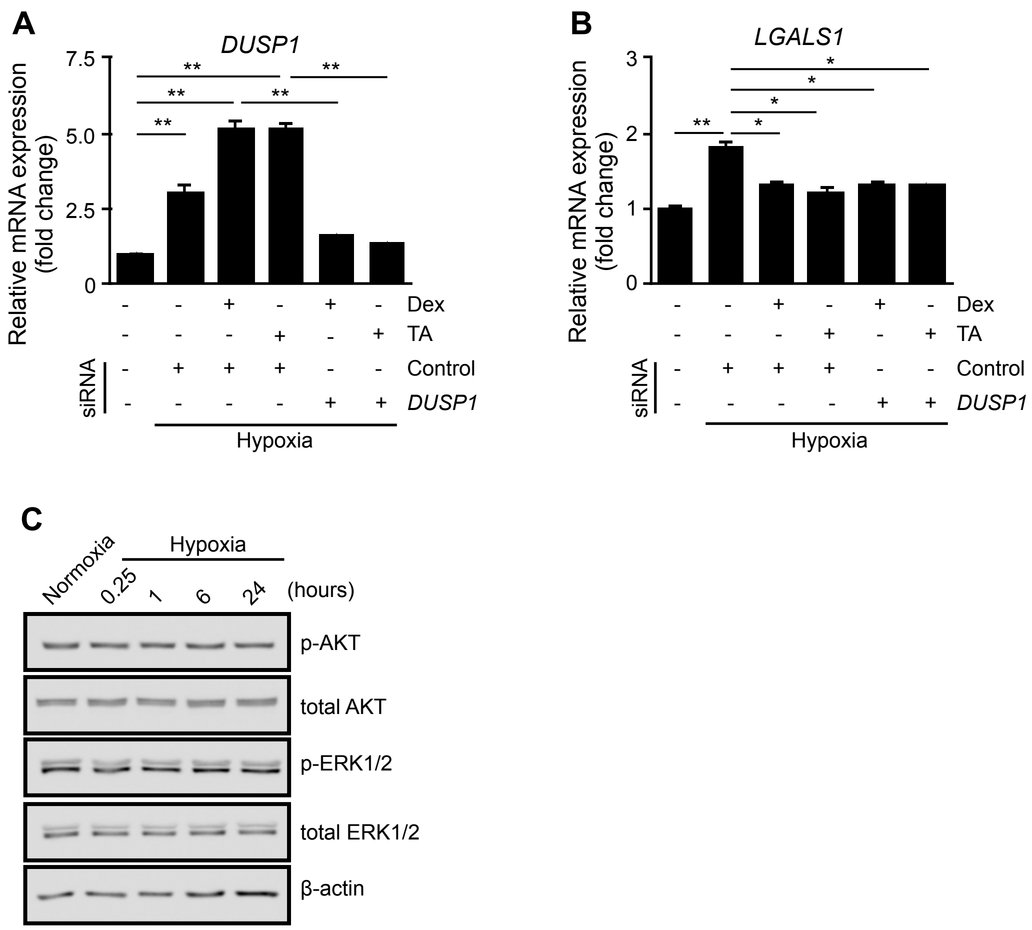

Supplementary Figure S2. Negligible impact of DUSP1 and its targeted signaling molecules on hypoxia-induced *LGALS1* expression

(A, B) *DUSP1* (A) and *LGALS1* (B) mRNA expression levels in human Müller glial cells exposed to control- or *DUSP1*-siRNA combined with dexamethasone (Dex, 1 μM) or triamcinolone acetonide (TA, 1 μM) for 30 minutes before culture in hypoxia (1% O_2) for 24 hours. (C) Müller glial cells were cultured in hypoxia (1% O_2) and harvested at the indicated times. Protein expression levels of phosphorylated and total forms of AKT and ERK1/2 were analyzed by immunoblot analysis. *p < 0.05, **p < 0.01, n = 4-6 per group.

Supplementary Figure 3

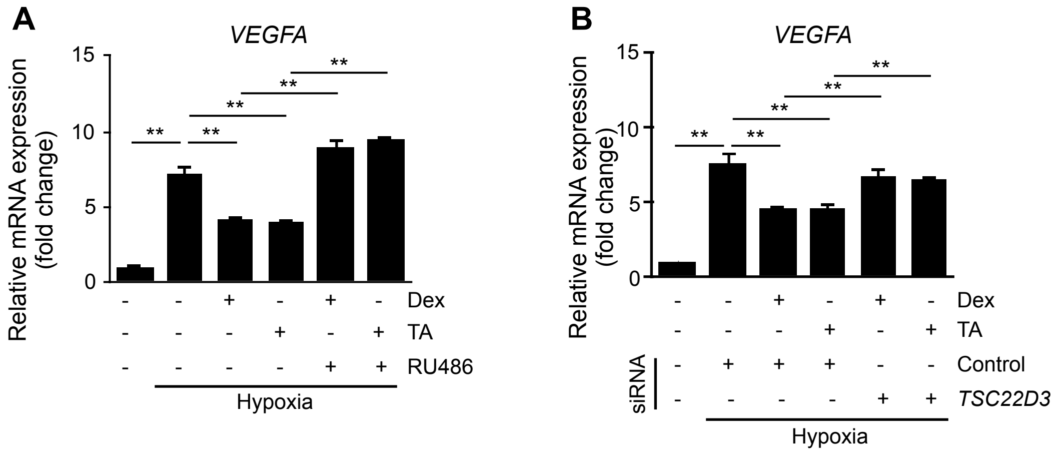

Supplementary Figure S3. TSC22D3-mediated suppression of hypoxia-induced *VEGFA* expression in Müller glial cells treated with glucocorticoids

(A) Müller glial cells were pretreated with the glucocorticoid receptor antagonist RU486 (1 μM) for 30 minutes before culture with Dex (1 μM) and TA (1 μM) in hypoxia (1% O_2) for 24 hours, and *VEGFA* gene expression levels were analyzed. (B) *VEGFA* mRNA expression levels in human Müller glial cells exposed to control- or *TSC22D3*-siRNA combined with Dex (1 μM) or TA (1 μM) for 30 minutes before culture in hypoxia (1% O_2) for 24 hours. $^{**}p < 0.01$, n = 6 per group.
